# Ascribing consciousness to artificial intelligence: human-AI interaction and its carry-over effects on human-human interaction

**DOI:** 10.3389/fpsyg.2024.1322781

**Published:** 2024-03-27

**Authors:** Rose E. Guingrich, Michael S. A. Graziano

**Affiliations:** ^1^Department of Psychology, Princeton University, Princeton, NJ, United States; ^2^Princeton School of Public and International Affairs, Princeton University, Princeton, NJ, United States; ^3^Princeton Neuroscience Institute, Princeton University, Princeton, NJ, United States

**Keywords:** artificial intelligence, human-AI interaction, theory of mind, consciousness, schemas, chatbots

## Abstract

The question of whether artificial intelligence (AI) can be considered conscious and therefore should be evaluated through a moral lens has surfaced in recent years. In this paper, we argue that whether AI is conscious is less of a concern than the fact that AI can be considered conscious by users during human-AI interaction, because this ascription of consciousness can lead to carry-over effects on human-human interaction. When AI is viewed as conscious like a human, then how people treat AI appears to carry over into how they treat other people due to activating schemas that are congruent to those activated during interactions with humans. In light of this potential, we might consider regulating how we treat AI, or how we build AI to evoke certain kinds of treatment from users, but not because AI is inherently sentient. This argument focuses on humanlike, social actor AI such as chatbots, digital voice assistants, and social robots. In the first part of the paper, we provide evidence for carry-over effects between perceptions of AI consciousness and behavior toward humans through literature on human-computer interaction, human-AI interaction, and the psychology of artificial agents. In the second part of the paper, we detail how the mechanism of schema activation can allow us to test consciousness perception as a driver of carry-over effects between human-AI interaction and human-human interaction. In essence, perceiving AI as conscious like a human, thereby activating congruent mind schemas during interaction, is a driver for behaviors and perceptions of AI that can carry over into how we treat humans. Therefore, the fact that people can ascribe humanlike consciousness to AI is worth considering, and moral protection for AI is also worth considering, regardless of AI’s inherent conscious or moral status.

## Introduction

Consciousness is considered the subjective experience that people feel in association with events, such as sensory events, memories, and emotions (Nagel, 1974; Harley, 2021). Many people study consciousness, and there are just as many competing theories about what it is and how it is generated in the human brain (e.g., Chalmers, 1996; Baars, 1997; Tononi, 2007; Graziano, 2013; Doerig et al., 2020). Recently, people have speculated that artificial intelligence can also have consciousness (e.g., O’Regan, 2012; Yampolskiy, 2018; Chalmers, 2023). Whether that is possible, and how, is still debated (e.g., Koch, 2019). However, it is undeniable that children and adults attribute consciousness to AI through Theory of Mind attributions (Kahn et al., 2012; Broadbent et al., 2013; Eyssel and Pfundmair, 2015; Martini et al., 2016; Tanibe et al., 2017; Świderska and Küster, 2018; Heyselaar and Bosse, 2020; Küster and Świderska, 2020; Taylor et al., 2020). Some researchers have argued that consciousness is fundamentally an attribution, a construct of social cognitive machinery, and that we attribute it to other people and to ourselves (Frith, 2002; Graziano, 2013; Prinz, 2017). As such, regardless of whether AI is conscious, attributing consciousness to AI matters in the same way attributing it to other humans does.

Premack and Woodruff (1978) coined the term Theory of Mind (ToM), which is the ability to attribute mind states to oneself and others more expansive. For example, one heavily studied aspect of ToM is the ability to recognize false beliefs in others (Wimmer and Perner, 1983). This cognitive capability has historically distinguished humans from many other species, yet Rabinowitz et al. (2018) claimed that artificial intelligence passed the false belief test. ToM may extend beyond attributing beliefs to attributing other aspects of mind such as emotions and intentionality. According to some, ToM can be divided into two distinct processes: attributing agency, or the ability to decide and act autonomously, and attributing experience, or the ability to have subjective states (Gray et al., 2007; Knobe and Prinz, 2007). Attributing consciousness to AI is therefore probably not one, single process, but instead should be broken down into experience and agency, with each part analyzed separately (Ward et al., 2013; Küster et al., 2020).

It has been suggested that attributing experience, rather than agency, plays a larger role in the perception of consciousness in AI (Knobe and Prinz, 2007). This distinction may present some difficulties for accurately measuring whether people view AI as conscious. People are generally more willing to assign agency rather than experience to a variety of targets, including robots (Gray and Wegner, 2012; Jacobs et al., 2021). This may be due in part to it being easier to determine whether an agent can make decisions or act on its own (agency) than whether an agent can feel pain or pleasure (experience). Adding further complexity, not all people ascribe agency and experience to AI in the same manner. For example, psychopathology and personality traits such as emotional stability and extraversion correlate with whether someone ascribes agency or experience to robots: emotional stability positively correlates with ascribing agency to robots, and extraversion positively correlates with attributing experience to robots (Tharp et al., 2016). Other individual differences such as people’s formal education may also relate to whether someone attributes agency characteristics like intentionality to a humanoid robot (Roselli et al., 2023). Given these findings, it may be useful to operationalize ToM as a complex, overarching collection of interrelated processes, each of which plays a different role in how people attribute consciousness to machines.

The attribution of consciousness to AI is particularly relevant to social actor AI. These humanlike agents are social embodiments of intelligent algorithms that people can talk to and even engage with physically. Social actor AI includes chatbots, digital voice assistants, and social robots. Social actor AI’s humanlike characteristics, from how the AI is embodied—like its bodily form, voice, and even linguistic style—to its ability to process social information, are unique within the category of artificial, non-human agents. Social actor AI is arguably more akin to humans than are other machines and objects. As such, how people behave toward social actor AI agents might be more likely to impact how they behave toward another human, despite the fact that these AI agents are not themselves living beings. Velez et al. (2019) posited that “an increasingly important question is how these social responses to agents will influence people’s subsequent interactions with humans.” Moreover, social actor AI is evolving rapidly. As Etzrodt et al. (2022) described it, “We are witnessing a profound change, in which communication *through* technologies is extended by communication *with* technologies.” Instead of using social media as a medium through which you can interact with other people, users can, for example, download an app through which they can interact with a non-human being. Companion chatbots like Replika, Anima, or Kiku have millions of people using their apps. Millions more have digital voice assistants such as Siri and Alexa operating on their smartphones and in their homes. People form relationships with these agents and can come to view them as members of the family, friends, and even lovers (Croes and Antheunis, 2020; Garg and Sengupta, 2020; Brandtzæg et al., 2022; Xie and Pentina, 2022; Guingrich and Graziano, 2023; Loh and Loh, 2023). AI agents will almost certainly become both more ubiquitous and humanlike. As new generations grow up with these technologies on their mobile devices and in their homes, the consequences of humanlike AI will likely become more pronounced over time.

In this paper, we will not consider what, exactly, consciousness is, what causes it, or whether non-human machines can have it. Instead, the goal here is to discuss how people perceive consciousness in social actor AI, to explore the possible profound social implications, and to suggest potential research questions and regulatory considerations for others to pursue within this scope of research.

## Part 1: evidence for carry-over effects between human-AI interaction and human-human interaction

### Carry-over effects between AI’s tangible and intangible characteristics

When people interact with AI, tangible characteristics of the agent such as appearance or embodiment, behavior, communication style, gender, and voice can affect how people perceive intangible characteristics such as mind and consciousness, emotional capability, trustworthiness, and moral status (Powers and Kiesler, 2006; Gray and Wegner, 2012; Broadbent et al., 2013; Eyssel and Pfundmair, 2015; Seeger and Heinzl, 2018; Lee et al., 2019; Küster et al., 2020; Dubosc et al., 2021; Rhim et al., 2022). The critical tangible-intangible relationship examined here is the one between an agent’s humanlike embodiment and consciousness ascription (Krach et al., 2008; Broadbent et al., 2013; Ferrari et al., 2016; Abubshait and Wiese, 2017; Stein et al., 2020).

Generally, the more tangibly humanlike that people perceive an AI agent to be, the more likely people are to ascribe mind to the agent (e.g., Broadbent et al., 2013). At least one study suggests that mind ascription does not increase with human likeness until a particular threshold of human likeness is reached; once an agent’s appearance reaches the middle of the machine-to-human spectrum and the AI agent’s appearance includes actual human features such as eyes and a nose, then mind ascription begins to increase with human likeness (Martini et al., 2016).

People are not always aware that they attribute mind to an AI agent during interaction. In other words, the construct of mind or consciousness activated in people during these interactions may be implicit, making it more difficult to measure. Banks (2019) conducted an online survey to compare participants’ implicit and explicit ascriptions of mind to an agent. Participants (*N* = 469) were recruited from social media and university research pools and were randomly assigned to one of four agents. Three of the agents were social AIs that varied in their human likeness and mind capacity, and one was a human control, all named “Ray.” Banks tested implicit ascription of mind using five classic ToM tests that measure whether participants ascribe mind to an agent including the white lie scenario and the Sally-Anne test. Explicit measures of mind were measured by two questions: do you think Ray has a mind, and how confident are you in your response? For the implicit tests’ open-ended responses, trained, independent raters coded the data for mentalistic explanations of behavior. The results showed that while people implicitly ascribed ToM to humanlike AI, this implicit ascription did not correlate with explicit mind ascriptions.

Mind ascription appears to be automatically induced by AI’s tangible human likeness, even when subjects are prompted to believe the opposite. Stein et al. (2020) compared mind ascriptions in a 2 × 2 between-subjects design of embodiment and mind capability for 134 German-speaking participants recruited from social media and mailing lists. Stimuli included vignettes and videos of either a text-based chatbot interface (Cleverbot) or a humanoid robot (with a 3-D rendered face of a woman) that was described as built on a simple or complex algorithm. The complex algorithm description included humanlike mind traits such as empathy, emotions, and understanding of the user. The researchers found a multivariate main effect of embodiment, such that people ascribed more mind capabilities to the humanoid robot than the text-based chatbot, regardless of whether it was based on a simple or complex algorithm. These researchers reported that “a digital agent with human-like visual features was indeed attributed with a more human-like mind—regardless of the cover story that was given regarding its actual mental prowess.”

In sum, evidence suggests that an AI agent’s observable or tangible characteristics, specifically its humanlike appearance, leads automatically to ascribing intangible characteristics, including consciousness, to the AI agent. As such, slight adjustments to AI’s tangible characteristics can impact whether people perceive the artificial agent as conscious.

### Carry-over effects between perceiving mind in AI and human-AI interaction

In some cases, ascribing a mind to AI is linked with viewing the agent as likable and trustworthy (Young and Monroe, 2019), which can impact whether people engage in helping behaviors. Srinivasan and Takayama (2016) found that when people perceived a robot as having an agentic mind, such that the robot was acting of its own accord rather than being controlled by a human, they came to its aid 50% more quickly. Study 1 was a mixed experiment design conducted online (*N* = 354, recruited from Amazon Mechanical Turk) in which participants each watched eight videos of robots requesting help using various politeness strategies, and study 2 was a behavioral lab study (*N* = 48, recruited via university participant pools and postings in local areas) with three conditions that were based on study 1’s results. In study 2, participants watched a movie with a robot in the room (Willow Garage’s Personal Robot 2). During the movie, the robot brought food to the participant and mentioned that the room looked like it needed to be cleaned, offered to do so, and requested aid from the participant. While the majority of participants helped the robot, those participants who rated the robot as more agentic came to its aid more quickly.

Depending on the paradigm, ascribing mind to AI can affect ease of interaction by augmenting or inhibiting the dyadic flow. Interacting with a humanlike artificial agent spurs the automatic use of human social scripts (Nass and Moon, 2000; Nass and Brave, 2005) and other social processes (von der Pütten et al., 2009), which can facilitate human-AI interaction (Sproull et al., 1996; Rickenberg and Reeves, 2000; Krämer et al., 2003a,b; Duffy, 2008; Krämer et al., 2009; Vogeley and Bente, 2010; Kupferberg et al., 2011). Facilitation of interaction and likability are however dependent on individual differences such as familiarity with the AI (Wang et al., 2021), need for social inclusion or interaction (Lee et al., 2006; Eyssel and Pfundmair, 2015), and other individual differences (Lee, 2010).

At a certain point, interaction facilitation no longer increases with human likeness across both tangible and intangible domains. The benefits of human likeness decrease dramatically when human likeness suddenly becomes creepy, according to the Uncanny Valley Hypothesis coined by Mori (1970). When an AI agent’s appearance approaches the tipping point of “not enough machine, not enough human,” the AI has entered the dip of the uncanny valley. At this point, an artificial agent’s human likeness becomes disturbing, thereby causing anxiety or discomfort in users. The discomfort arising from the uncanny valley effect is generally distinct from dislike yet can have similar negative effects on the flow of interaction (Quadflieg et al., 2016).

The uncanny valley theory of human-AI interaction more recently acquired a qualifier: the uncanny valley of *mind* (Stein and Ohler, 2017; Appel et al., 2020). No longer just concerned with general human likeness, the uncanny valley effect can occur when AI’s mind capabilities get too close to that of a human mind. It is uncertain whether negative uncanny valley effects of mind are stable, however, given the contradictions within this more recent scope of research. In Stein et al.’s study, they also found that the AI with low mind capacity, based on a simple algorithm rather than an advanced one, caused more discomfort when the AI was embodied rather than solely text-based. In another study, the researchers found that the more people perceived AI *or* humans to have a typically human mind, the less eerie feelings they experienced (Quadflieg et al., 2016). Due to inconsistent stimuli across studies, it is possible that slight variations in facial features or voice of the AI agent drove these dissimilar effects. In these cases, it may be useful to control for appearance when attempting to parse out the impacts of the uncanny valley of mind on how people interact with AI agents.

Via a series of three studies, Gray and Wegner (2012) made the claim that experiential aspects of mind, and not those of agentic mind, drive uncanny valley effects. In one of the studies, participants, recruited from subway stations and dining halls (*N* = 45), were given vignettes of a supercomputer that was described as having only experience capabilities, having only agency, or simply mechanical. They then rated their feelings (uneasy, unnerved, and creeped out) and perceptions of the supercomputer’s agency and experience. The experiential supercomputer elicited significantly higher uncanny valley feelings than agents in the other two conditions. Apparently, an intelligent computer that is seen as having emotion is creepier than one that can make autonomous decisions. The distinction between uncanny valley effects of experience and agency may be caused by feelings of threat: AI agents that are capable of humanlike emotion threaten that which makes mankind special (Stein and Ohler, 2017). If threat drove discomfort in Gray and Wegner’s participants, then familiarity with the agent might mitigate perceptions of threat to the point at which the uncanny valley switches into the “happy valley.” According to that hypothesis, after long-term, comfortable, and safe exposure to a humanlike AI agent, people might find the agent’s human likeness to increase its likability, which might facilitate human-AI interaction (Cheetham et al., 2014).

The uncanny valley effect with respect to AI is therefore more complicated and difficult to study than it may at first appear. Familiarity with AI over time, combined with the increasing ubiquity of social actor AI, may eliminate uncanny valley effects altogether. Uncanny valley effects differ across studies, and are affected by multiple factors, including expectation violation (Spence et al., 2014; Edwards et al., 2019; Lew and Walther, 2022), individual differences (MacDorman and Entezari, 2015), and methodological differences such as stimuli and framing. Further, the way the uncanny valley graph rises to a peak has been contested. For example, researchers have debated exactly where that peak lies on the machine-to-human scale (Cheetham et al., 2014; Pütten and Krämer, 2014; Stein et al., 2020). However, what we do know is that perceiving mind in AI affects people’s emotional state and how they interact with AI, making the intangible characteristic of mind one of the mechanisms that impacts human-AI interaction.

### Carry-over effects between human-AI interaction and human-human interaction

Most studies on human-AI interactions, such as those reviewed above, focus on what could be called one-step effects like the uncanny valley effect, trust, and likability. Such studies are concerned with how characteristics of AI impact how people interact with the agent. Arguably a more important question is the two-step effect of how human-AI interactions might impact subsequent human-human interactions. Though findings on these two-step effects are limited and sometimes indirect, the data do suggest that such effects are present. The impact of AI is not confined to the interaction between a user and an AI agent, but rather carries over into subsequent interactions between people.

Social Cognitive Theory, anthropomorphism, and ToM literature provide theoretical foundations for why interactions with social actor AI could prompt carry-over effects on human-human interaction. Due to the social nature of these agents, AI can act as a model for social behavior that users may learn from (Bandura, 1965, 1977). According to Waytz et al. (2010), when someone anthropomorphizes or ascribes mind to an artificial agent, that agent then “serves as a source of social influence on the self.” In other words, “being watched by others matters, perhaps especially when others have a mind like one’s own.” Social actor AI is an anthropomorphized target; therefore, it can serve as a role model or operate as an ingroup member that has some involvement in setting social norms, as seen with the persuasive chatbot that convinced people to donate less to charity (Zhou et al., 2022), the chatbot that persuaded users to get vaccinated for COVID-19 or participate in social distancing (Kim and Ryoo, 2022), and the humanlike avatar that elicited more socially desirable responses from participants than a mere text-based chatbot did (Krämer et al., 2003a). Social actor AI can persuade people in these ways, regardless of whether people trust it or perceive it as credible (Lee and Liang, 2016, 2019). In some paradigms, chatbot influence mimics that of people: chatbots can implement foot-in-the-door techniques to influence people’s emotions and bidding behavior in gambling (Teubner et al., 2015) and can alter consumers’ attitudes and purchasing behavior (Han, 2021; Poushneh, 2021).

Another explanation for why AI can socially influence people may be that the user views the agent as being controlled by another human. Some research suggests that perceiving a human in the loop during interactions with AI results in stronger social influence and more social behavior (Appel et al., 2012; Fox et al., 2014). This idea, however, has since been contested (Krämer et al., 2015). Indeed, early research on human-computer interaction found that when people perceived a computer as a social agent, they did not simply view it as a product of human creation, nor did they imagine that they were interacting with the human engineer who created the machine (Nass et al., 1994; Sundar and Nass, 2000). Nass and colleagues designed a series of paradigms in which participants were tutored, via audio emitting from computer terminals, by computers or human programmers that subsequently evaluated participants’ performance. To account for the novelty of computers at this time, earlier studies were conducted with experienced computer users. They found significant differences between computer and human tutor conditions, such that people viewed computers as not just entities controlled by human programmers, but entities to which the ideas of “self” and “other” and social agency applied. Nass and colleagues laid the groundwork for evaluating social consequences of interacting with intelligent machines, as their experiments provided initial evidence that people treated the machines themselves as social actors. As such, it may be the case that social influence is strengthened when people think a human is involved, yet social influence still exists when the AI agent is perceived as acting on its own accord.

Communication researchers have found that the way people communicate with AI is linked to how they communicate with other humans thereafter, such that people are then more likely to speak to another human in the same way in which they habitually speak to an artificial agent. For example, talking with the companion chatbot Replika caused users’ linguistic styles to converge with the style of their chatbot over time (Wilkenfeld et al., 2022). The way children speak with social actor AI such as the home assistant, Alexa, can carry over into how children speak to their parents and others (Hiniker et al., 2021). Garg and Sengupta (2020) tracked and interviewed 18 families over an average of 58 weeks who used a digital voice assistant in their homes and analyzed raw audio interactions with their assistant. These researchers found that “when children give commands at a high volume, there is an aggressive tone, which often unintentionally seeps into children’s conversations with friends and family.” A parent in the study commented that, “If I do not listen to what my son is saying, he will just start shouting in an aggressive tone. He thinks, as Google responds to such a tone, I would too.” While home assistants can negatively impact communication, they can also foster communication within families and alter how communication breakdowns are repaired (Beneteau et al., 2019, 2020). Parents have concerns about their children interacting with social actor AI, but they also see AI’s potential to support children by “attuning to others, cultivating curiosity, reinforcing politeness, and developing emotional awareness” (Fu et al., 2022). According to the observational learning concept in Social Cognitive Theory (Bandura, 1965), assistants might provide models for prosocial behavior that children could learn from (such as being polite, patient, and helpful) regardless of whether the assistant provides positive reinforcement when children act in these prosocial ways. The studies mentioned above show how both children’s positive and negative modes of communication can be reinforced via interactions with home assistants.

Not only can social actor AI affect the way that people communicate with each other within their relationships, but also it has the potential to impact relationships with other people due to attachment to the agent. Through in-depth interviews of existing Replika users (*N* = 14, ages 18–60), Xie and Pentina (2022) suggested that AI companions might replace important social roles such as family, friends, and romantic partners through unhealthy attachment and addiction. An analysis of families’ use of Google Home revealed that children, specifically those between the age of 5–7, believed the device to have feelings, thoughts, and intentions and developed an emotional attachment to it (Garg and Sengupta, 2020). These children viewed Google Home as if it had a mind through ascribing characteristics of agency and experience to it.

The psychosocial benefits of interactions with social actor AI may either contribute to positive relational skill-building if AI is used as a tool, or they may lead to human relationship replacement if these benefits are comparatively too difficult to get from relationships with real people. Research suggests that people self-disclose more when interacting with a computer versus with a real person, in part due to people having lower fear of being judged, thereby prompting more honest answers (Lucas et al., 2014). This effect is found even though benefits of emotional self-disclosure are equal whether people are interacting with chatbots or human partners (Ho et al., 2018). Further, compared to interacting with other people, those interacting with artificial agents experience fewer negative emotions and lower desire for revenge or retaliation (Kim et al., 2014). Surveys of users of the companion chatbot, Replika, suggest that users find solace in human-chatbot relationships. Specifically, those who have experienced trauma in their human relationships, for example, indicate that Replika provides a safe, consistent space for positive social interaction that can benefit their social health (Ta et al., 2020; Guingrich and Graziano, 2023). The question is whether the benefits of human-AI interaction presented here may lead to people choosing AI companions over human ones.

In part 1, we have reviewed evidence that human-AI interaction, when moderated by perceiving the agent as having a humanlike mind or consciousness, has carry-over effects on human-human interaction. In part 2, we address the mechanism of this moderator through congruent schema activation. We further pose two theoretical types of carry-over effects that may occur via congruent schema activation: relief and practice.

## Part 2: mechanisms and types of carry-over effects: schemas and relief or practice

### Schema congruence and categorization

What is the mechanism by which people’s attributions of consciousness to AI lead to carry-over effects on interactions with other humans? One possibility is the well-known mechanism of activating similar schemas of mind when interacting with different agents. We propose that ascribing mind or consciousness to AI through automatic, congruent schema activation is the driving mechanism for carry-over effects between human-AI interaction and human-human interaction.

Schemas are mental models with identifiable properties that are activated when engaging with an agent or idea and are useful ways of organizing information that help inform how to conceptualize and interact with new stimuli (Ortony and Anderson, 1977; McVee et al., 2005; Pankin, 2013). For example, the schema you have for your own consciousness informs how you understand the consciousness of others. You assume, because your experience of consciousness contains X and Y characteristics, that another person’s consciousness also contains X and Y characteristics, and this facilitates understanding and subsequent social interaction between you and the other person (Graziano, 2013).

Researchers have analyzed the consequences of failing to fully activate all properties of mind schemas between similar agents. For example, the act of dehumanization reflects a disconnect between how you view your mind and that of other people. Instead of activating the consciousness schema with X and Y characteristics during interaction with another human, you may activate only the X characteristic of the schema. Dehumanization is linked to social consequences such as ostracism and exclusion, which can harm social interaction (Bastian and Haslam, 2010; Haslam and Loughnan, 2014).

We can apply the idea of schema congruence to interactions with social actor AI while also taking into consideration the level of advancement of the AI in question. Despite AI being more advanced than other technology like personal mobile devices or cars in terms of human likeness and mind ascription, some research suggests that social actor AI still falls short of the types of mind schemas that are activated when people interact with each other. However, humanlike AI is developing at a rapid rate. As it does, the schematic differences between AI agents and humans will likely blur more than they already have. To better understand the consequences of current social actor AI, it may be prudent to observe the impacts of human-AI interaction through ingroup-outgroup or dehumanization processes, both of which are useful psychological lenses for group categorization. We propose that psychological tests of mind schema activation will be especially useful for more advanced, future AI that is more clearly different from possessions like cars and phones but similar to humans in terms of mind characteristics.

### Schematic incongruence yields uncanny valley effects

Categorization literature attempts to delineate whether people treat social actor AI as non-human, human, or other. The data are mixed, but some of the results may stem from earlier AI that is not as capable. Now that AI is becoming sophisticated enough that people can more easily attribute mind to it, the categories may change. In this literature, social AI is usually classified by study participants as somewhere on the spectrum between machine and human, or it is classified as belonging to its own, separate category (Severson and Carlson, 2010). That separate category is often described as not quite machine, not quite human, with advanced communication skills and other social capabilities, and has been labeled with mixed-category words like humanlike, humanoid, and personified things (Etzrodt and Engesser, 2021).

Some researchers claim that the uncanny valley effect is driven by categorization issues. In that hypothesis, humanlike AI is creepy because it does not fit into categories for machine or human but exists in a space for which people do not have a natural, defined category (Burleigh et al., 2013; Kätsyri et al., 2015; Kawabe et al., 2017). Others claim that category uncertainty is not the driver of the uncanny valley effect, but, rather, inconsistency is (MacDorman and Chattopadhyay, 2016). In that hypothesis, because of the inconsistencies between AI and the defining features of known categories, people treat humanoid AI agents as though they do not fit into a natural, existing category (Gong and Nass, 2007; Kahn et al., 2011). Because social actor AI defies boundaries, it may trigger outgroup processing effects such as dehumanization that contribute to negative affect. The cognitive load associated with category uncertainty, more generally, may also trigger negative emotions that are associated with the uncanny valley effect.

Social norms likely play a role in explicit categorization of social AI (Hoyt et al., 2003). People may be adhering to a perceived social norm when they categorize social AI as machinelike rather than humanlike. It is possible that people explicitly place AI into a separate category from people, while the implicit schemas activated during interaction contradict this separation. The uneasy feeling from the uncanny valley effect may be a product of people switching between ascribing congruent mind schemas to the agent in one moment and incongruent ones in the next.

### Schematic congruence yields carry-over effects on human-human interaction

As humanlike AI approaches the human end of the machine-to-human categorization spectrum, it also advances toward a position in which people can more easily ascribe a conscious mind to it, thereby activating congruent mind schemas during interactions with it. Activating congruent schemas impacts how people judge the agent and its actions. For example, the belief that you share the same phenomenological experience with a robot changes the way you view its level of intent or agency (Marchesi et al., 2022). Activation of mind-similarity may resemble simulation theory (Harris, 1992; Röska-Hardy, 2008). In that hypothesis, the observer does not merely believe the artificial agent has a mind but simulates that mind through the neural machinery of the person’s own mind. Simulation allows the agent to seem more familiar, which facilitates interaction.

Some researchers have used schemas as a lens to explain why people interact differently with computer partners vs. human ones (Hayashi and Miwa, 2009; Merritt, 2012; Velez et al., 2019). In this type of research, participants play a game online and are told that their teammate is either a human or a computer, but, unbeknownst to the participants, they all interact with the same confederate-controlled player. This method allows researchers to observe how schemas drive perceptions and behavior, given that the prime is the only difference. According to Fox et al. (2014), when people believed themselves to be interacting with a human agent, they were more likely to be socially influenced. Velez et al. (2019) took this paradigm one step further and observed that activating schemas of a human mind during an initial interaction with an agent resulted in carry-over effects on subsequent interactions with a human agent. These researchers employed a 2 × 2 between-subjects design in which participants played a video game with a computer agent or human-backed avatar. They then were presented with the option to engage prosocially through a prisoner’s dilemma money exchange with a stranger thereafter. When participants (*N* = 184) thought they were interacting with a human and that player acted pro-socially, they behaved more pro-socially toward the stranger. However, when participants believed they were interacting with a computer-controlled agent and it behaved pro-socially toward them, they had lower expectations of reciprocity and donated less game credits to the human stranger with whom they interacted subsequently. In the interpretation of Velez et al., the automatic anthropomorphism of the computer-backed agent was a mindless process (Kim and Sundar, 2012) and therefore not compatible with the cognitive-load-requiring social processes thereafter (Velez et al., 2019).

One of the theories that arose from research on schema activation in gaming is the Cooperation Attribution Framework (Merritt, 2012). According to Merritt, the reason people behave differently when game playing with a human vs. an artificial partner is that they generate different initial expectations about the teammate. These expectations activate stereotypes congruent with the teammates’ identity, and confirmations of those stereotypes are given more attention during game play, causing a divergence in measured outcomes. According to Merritt, “the differences observed are broadly the result of being unable to imagine that an AI teammate could have certain attributes (e.g., emotional dispositions). …the ‘inability to imagine’ impacts decisions and judgments that seem quite unrelated.” The computer-backed agents used in this research may evoke a schema incompatible with humanness—one that aligns with the schema of a pre-programmed player without agency—whereas more modern, advanced AI might evoke a different, more congruent schema in human game players.

Other studies examined schema congruence by seeing how people interact with and perceive an AI agent if its appearance and behavior do not fit into the same humanlike category. Expectation violation and schema incongruence appear to impact social responses to AI agents. In two studies, Ciardo et al. (2021, 2022) manipulated whether an AI agent looked humanlike and made errors in humanlike (vs. mechanical) ways. They then observed whether people attributed intentionality to the agent or were socially inclusive with it. Coordination with the AI agent during the task and social inclusion with the AI agent after the task were impacted by humanlike errors during the task only if the agent’s appearance was also humanlike. This variation in response toward the AI may have to do with ease of categorization: if an agent looks humanlike and acts humanlike, the schemas activated during interaction are stable, which facilitates social response to the agent. On the other hand, if an agent looks humanlike but does not act humanlike, schemas may be switching and people may incur cognitive load and feel uncertain about how to respond to the agent’s errors. In their other study, these researchers found that when a humanlike AI agent’s mistakes were also humanlike, people attributed more intentionality to it than when a humanlike AI agent’s mistakes were mechanical.

To understand why people might unconsciously or consciously view social actor AI as having humanlike consciousness, it is useful to understand individual differences that contribute to automatic anthropomorphism (Waytz et al., 2010) and therefore congruent schema activation. Children who have invisible imaginary friends are more likely to anthropomorphize technology, and this is mediated by what the researchers call the “imaginative process of simulating and projecting internal states” through role-play (Severson and Woodard, 2018). As social AI agents become more ubiquitous, it is likely that mind-ascription anthropomorphism will occur more readily; for instance, intensity of interaction with the chatbot Replika mediates anthropomorphism (Pentina et al., 2023). Currently, AI is not humanlike enough to be indistinguishable from real humans. People are still able to identify real from artificial at a level better than chance, but this is changing. What might happen once AI becomes even more humanlike to the point of being indistinguishable from real humans? At that point, the people who have yet to generate a congruent consciousness schema for social actor AI may do so. Others may respond by becoming more sensitive to subtle, distinguishing cues and by creating more distinct categories for humans and AI agents. At some point in the development of AI, perhaps even in the near future, the distinction between AI behavior and real human behavior may disappear entirely, and it may become impossible for people to accurately separate these categories no matter how sensitive they are to the available cues.

### Possible types of carry-over effects: relief or practice

What, exactly, is the carry-over effect between human-AI interaction and human-human interaction? We will examine two types of carry-over effects that do not necessarily reflect all potential outcomes but that provide a useful comparison by way of their consequences: relief and practice. In the case of relief, doing X behavior with AI will cause you to do less of X behavior with humans subsequently. In the case of practice, doing X behavior with AI will cause you to do more of X behavior with humans subsequently. The preponderance of the evidence so far suggests that practice is more likely to be observed, and its consequences outweigh those of relief (Garg and Sengupta, 2020; Hiniker et al., 2021; Wilkenfeld et al., 2022).

The following scenarios illustrate theoretical examples of both effects. Consider an example of relief. You are angry, and you let out your emotions on a chatbot. Because the chatbot has advanced communication capabilities and can respond intelligently to your inputs, you feel a sense of relief from berating something that reacts to your anger. Over time, you rely on ranting to this chatbot to release your anger, and as a result, you are relieved of your negative emotions and are less likely to lash out at other people.

Now consider an example of practice. Suppose you are angry. You decide to talk to a companion chatbot and unleash your negative emotions on the chatbot, speaking to it rudely through name-calling and insults. The chatbot responds only positively or neutrally to your attacks, offering no negative backlash in return. This works for you, so you continue to lash out at the chatbot when angry. Since this chatbot is humanlike, you tend not to distinguish between this chatbot and other humans. Over time, you start to lash out at people as well, since you have not received negative feedback from lashing out at a humanlike agent. The risk threshold for relieving your anger at something that will socialize with you is decreased. You have effectively practiced negative behavior with a humanlike chatbot, which led to you engaging more in that type of negative behavior with humans. Practice can involve more than negative behaviors. Suppose you have a friendly, cooperative interaction with an AI, in which you feel safe enough to share your feelings. Having engaged in that practice, maybe you are more likely to engage in similar positive behavior to others in your life.

Both of these examples illustrate ways in which antisocial behavior toward humans can be reduced or increased by interactions with social actor AI. There are also situations in which prosocial behaviors can be reinforced. Which of the scenarios, relief or practice, are we more likely to observe? The answer to this question will inform the way society should respond to or regulate social actor AI.

### Evidence against relief and evidence for practice effects

Researchers have proposed that people should take advantage of social actor AI’s human likeness to use it as a cathartic object. Coined by Luria et al. (2020), the idea of a cathartic object is familiar: for example, a pillow can be used as a cathartic object by punching it in anger, thereby relieving oneself of the emotion. This is, colloquially, a socially acceptable behavior toward the target. Luria takes this one step further by suggesting that responsive, robotic agents that react to pain or other negative input can provide even more relief than an inanimate object, and that we should use them as cathartic objects. Luria claims that the reaction itself, which mirrors a humanlike pain response, provides greater relief than that of an object that does not react. One such “cathartic object” designed by Luria is a cushion that vibrates in reaction to being poked by a sharp tool. The more tools you put into the cushion, the more it vibrates until it shakes so violently that the tools fall out. You can repeat the process as much as desired.

The objects presented by Luria as potential agents of negative-emotion relief are simply moving, responsive objects at this stage. However, Luria proposes the use of more humanlike agents, such as social robots, as cathartic objects. In one such proposition, Luria suggests that people throw knives at a robotic, humanlike bust that responds to pain. In another example, Luria suggests a ceremonial interaction in which a child relieves negative emotions with a responsive robot that looks like a duck.

Luria’s proposal rests on the assumption that releasing negative emotions on social robots will relieve the user of that emotion. Catharsis literature, however, challenges this assumption: research suggests that catharsis of aggression does not reduce subsequent aggression, but can in fact increase it, providing evidence for practice effects (Denzler and Förster, 2012; Konečni, 2016). Catharsis researchers posit that the catharsis of negative behavior and feelings requires subsequent training, learning, and self-development post-catharsis to lead to a reduction of the behavior. Therapy, for example, provides a mode through which patients can feel catharsis and then learn methods to reduce negative feelings or behaviors toward others. Even so, the catharsis or immediate relief alone does not promise a reduction of that behavior or feeling (Alexander and French, 1946; Dollard and Miller, 1950; Worchel, 1957) and can in many ways exacerbate negative feelings (Anderson and Bushman, 2002; Bushman, 2002). Other researchers found that writing down feelings of anger was less effective than writing to the person who made the participant angry, yet neither mode of catharsis alleviated anger responses (Zhan et al., 2021). These findings suggest that whether you were to write to a chatbot and tell it about your anger, or bully it, the behavior would only result in increased aggression toward other people.

Recent data on children and their interactions with home assistants such as Amazon’s Alexa or Google Assistant suggest for plural data that negative interactions with AI, including using an aggressive, loud tone of voice with it, does not lead to a cathartic reduction in aggression toward others, but to the opposite, an increase in aggressive tone toward other people (Beneteau et al., 2019, 2020; Garg and Sengupta, 2020; Hiniker et al., 2021). This data suggests that catharsis does not work for children in their interactions with AI and may be cause for concern.

This concern is especially important given that children tend to perceive a humanlike mind in non-human objects in general, more so than adults. When asked to distinguish between living and non-living agents, including robots, children experience some difficulty. Even when children do not ascribe biological properties to robots, research suggests that children can still ascribe psychological properties, like agency and experience, to robots (Nigam and Klahr, 2000). There appears to be a historical trend of increasing mind ascription to technology in children over the years. This trend may reflect the increased human likeness and skills of technology, and therefore provide us a prediction for the future. In 1995, children at the age of five reported that robots and computers did not have brains like people (Scaife and Van Duuren, 1995), but in a research study in 2000, children ascribed emotion, cognitive abilities, and volition to robots, even though most did not consider the robot to be alive (Nigam and Klahr, 2000). In studies conducted in 2002 and 2003, children 3–4 years old tended not to ascribe experiential mind to robots but did ascribe agentic qualities such as the ability to think and remember (Mikropoulos et al., 2003). According to Severson and Woodard (2018), not unlike some theories of consciousness in which people perceive there to be a person inside their mind, “There are numerous anecdotes that young children think there’s a little person inside the device” in home assistants like Alexa. Children with more exposure to and affinity with digital voice assistants have more pronounced psychological conceptions of technology, but it is unclear whether conceptions of technology and living things are blurred together (Festerling et al., 2022). Children do distinguish between technology and other living things through ascriptions of intelligence, however (Bernstein and Crowley, 2008). Goal-directed, autonomous behavior (a component of ToM) is one of the key mechanisms by which children distinguish an object as being alive (Opfer, 2002; Opfer and Siegler, 2004). Given that children appear to be ascribing mind to technology more than ever, this trend is likely to continue with AI advancement.

We are skeptical that socially mistreating AI can result in emotional relief, translating into better social behavior toward other people. Although the theory has been proposed, little if any evidence supports it. Encouraging people, and especially children, to berate or socially mistreat AI on the theory that it will help them become kinder toward people seems ill-advised to us. In contrast, the existing evidence suggests that human treatment of AI can sometimes result in a practice effect, which carries over to how people treat each other. Those practice effects could either result in social harm, if antisocial behavior is practiced, or social benefit, if pro-social behavior is practiced.

## Discussion

### The moral issue of perceiving consciousness in AI and suggested regulations

As stated at the beginning of this article, we do not take sides here on the question of whether AI is conscious. However, we argue that the fact that people often perceive it to be conscious is important and has social consequences. Mind perception is central to this process, and mind perception itself evokes moral thinking. Some researchers claim that “mind perception is the essence of morality” (Gray and Wegner, 2012). When people perceive mind in an agent, they may also view it as capable of having conscious experience and therefore perceive it as something worthy of moral care (Gray et al., 2007). Mind perception moderates whether someone judges an artificial agent’s actions as moral or immoral (Shank et al., 2021). We suggest that when people perceive an agent to possess subjective experience, they perceive it to be conscious; when they perceive it to be conscious, they are more likely to perceive it as worthy of moral consideration. A conscious being is perceived as an entity that can act morally or immorally, and that can be treated morally or immorally.

We suggest it is worth at least considering whether social actor AI, as it becomes more humanlike, should be viewed as having the status of a moral patient or a protected being that should be treated with care. The crucial question may not be whether the artificial agent deserves moral protection, but rather whether we humans will harm ourselves socially and emotionally if we practice harming humanlike AI, and whether we will help ourselves if we practice pro-social behavior toward humanlike AI. We have before us the potential for cultural improvement or cultural harm as we continue to integrate social actor AI into our world. How can we ensure that we use AI for good? There are several options, some of which are unlikely and unenforceable, and one of which we view as being the optimal choice.

One option is to enforce how people treat AI, to reduce the risk of the public practicing antisocial behavior and to increase the practice of prosocial behavior. Some have taken the stance that AI should be morally protected. According to philosophers such as Ryland (2021a,b), who characterizes relationships with robots in terms of friendship and hate, hate toward robots is morally wrong, and we should consider it even more so as robots become more humanlike. Others have claimed that we should give AI rights or protections, because AI inherently deserves them due to its moral-care status (Akst, 2023). Not only is this suggestion vague, but it is also pragmatically unlikely. Politically, it is overwhelmingly unlikely that any law would be passed in which a human being is supposed to be arrested, charged, or serve jail time for abusing a chatbot. The first politician to suggest it would end their career. Any political party to support it would lose the electorate. We can barely pass laws to protect transgender people; imagine the political and cultural backlash to any such legal protections for non-human machines. Regulating human treatment of AI is, in our opinion, a non-starter.

A second option is to regulate AI such that it discourages antisocial behavior and encourages prosocial behavior. We suggest this second option is much more feasible. For example, abusive treatment of AI by the user could be met with a lack of response (the old, “just ignore the bully and he’ll go away, because he will not get the reaction he’s looking for”). The industries backing digital voice assistants have already begun to integrate this approach into responses to bullying speech. In 2010, if a user told Siri, “You’re a slut,” it was programmed to respond with, “I’d blush if I could.” Due to stakeholder feedback, the response has now been changed to a more socially healthy, “I will not respond to that” (UNESCO & EQUALS Skills Coalition et al., 2019; UNESCO, 2020). Currently, the largest industries backing AI, such as OpenAI with ChatGPT, are altering and restricting the types of inputs their social actor AI will respond to. This trend toward industry self-regulation of AI is encouraging. However, we are currently entirely dependent on the good intentions of industry leaders to control whether social actor AI encourages prosocial or antisocial behavior in users. Governing bodies have begun to make regulation attempts, but their proposals have received criticism: such documents try a “one-size-fits-all approach” that may result in further inequality. For example, the EU drafted an Artificial Intelligence Act (AIA) that proposes a ban on AI that causes psychological harm, but the potential pitfalls of this legislation appear to outweigh its impact on psychological well-being (Pałka, 2023).

Social actor AI is increasingly infiltrating every part of society, interacting with an increasing percentage of humanity, and therefore even if it only subtly shapes the psychological state and interpersonal behavior of each user, it could cause a massive shift of normative social behavior across the world. If there is to be government regulation of AI to reduce its risk and increase its benefit to humanity, we suggest that regulations aimed at its prosociality would make the biggest difference. One could imagine a Food and Drug Administration (FDA) style agency, informed by psychological experts, that studies how to build AI such that it reinforces prosociality in users. Assays could be developed to test AI on sample groups to measure its short- and long-term psychological impacts on users, data that is unfortunately largely missing at the present time. Perhaps, akin to FDA regulations on new drugs, new AI that is slated to be released to a wider public should be put through a battery of tests to show that, at the very least, it does no psychological harm. Drug companies are required to show extensive safety data before releasing a product. AI companies currently are not. It is in this space that government regulation of AI makes sense to us.

Others have made claims in the name of ethics about regulating characteristics of AI; however, these suggestions seem outdated. According to Bryson (2010), robots should be “slaves”—this does not mean that we should make robots slaves, but rather, we should keep them at a simpler developmental level by not giving them characteristics that might enable people to view them as anything other than owned and created by humans for humans. Bryson claims that it would be immoral to create a robot that can feel emotions like pain. Metzinger (2021) called for a ban on development of AI that could be considered sentient. AI advancement, however, continues in this direction. Calls for stopping the technological progress have not been effective. Relatively early in development of social actor AI, computer science researchers created benchmarks for human likeness to enable people to create more humanlike AI (Kahn et al., 2007). That human likeness has increased since. Our proposal has less to do with regulating how advanced or how humanlike AI becomes, and more to do with regulating how AI impacts the psychology of users by providing a model for prosocial behavior or by ignoring, confronting, or rectifying antisocial behavior.

Almost all discussion of regulating AI centers around its potential for harm. We will end this article by noting the enormous potential for benefit, especially in light of AI’s guaranteed permanence in our present and future. Social AI is increasingly similar to humans in that it can engage in humanlike discourse, appear humanlike, and impact our social attitudes and interactions. Yet, social AI differs from humans in at least one significant way: it does not experience social or emotional fatigue. The opportunity to practice prosocial behavior is endless. For example, a chatbot will not grow tired and upset if you need to constructively work through a conflict with it. Neither will a chatbot disappear in the middle of a conversation when you are experiencing sadness or hurt and are in need of a friend. Social actor AI can both provide support and model prosocial behavior by remaining polite and present. Chatbots like WoeBot help users work through difficult issues by asking questions in the style of cognitive behavioral therapy (Fitzpatrick et al., 2017). Much like the benefits of journaling (Pennebaker, 1997, 2004), this human-chatbot engagement guides the user to make meaning of their experiences. It is worth noting that people who feel isolated or have experienced social rejection or social frustration may be a significant source of political and social disruption in today’s world. If a universally available companion bot could boost their sense of social well-being and allow them to improve their social interaction skills through practice, that tool could make a sizable contribution to society. If AI is regulated such that it encourages people to treat it in a positive, pro-social way, and if carry-over effects are real, then AI becomes a potential source of enormous social and psychological good in the world.

If we are to effectively tackle the ever-growing issue of what to do in response to the surge of AI in our world, we cannot continue to point out only the ways in which it is harmful. AI is here to stay, and therefore we should be pragmatic with our approach. By understanding the ways in which interactions with AI can be both positive and negative, we can start to mitigate the bad by replacing it with the good.

## Data availability statement

The original contributions presented in the study are included in the article/supplementary material, further inquiries can be directed to the corresponding author.

## Author contributions

RG: Conceptualization, Funding acquisition, Investigation, Resources, Validation, Writing – original draft, Writing – review & editing. MG: Funding acquisition, Project administration, Resources, Supervision, Writing – review & editing.
